# Inhibition of Oxidative Neurotoxicity and Scopolamine-Induced Memory Impairment by *γ*-Mangostin: *In Vitro* and *In Vivo* Evidence

**DOI:** 10.1155/2019/3640753

**Published:** 2019-03-24

**Authors:** Youngmun Lee, Sunyoung Kim, Yeonsoo Oh, Young-Mi Kim, Young-Won Chin, Jungsook Cho

**Affiliations:** College of Pharmacy, Dongguk University-Seoul, Goyang, Gyeonggi-do 10326, Republic of Korea

## Abstract

Among a series of xanthones identified from mangosteen, the fruit of *Garcinia mangostana* L. (Guttifereae), *α*- and *γ*-mangostins are known to be major constituents exhibiting diverse biological activities. However, the effects of *γ*-mangostin on oxidative neurotoxicity and impaired memory are yet to be elucidated. In the present study, the protective effect of *γ*-mangostin on oxidative stress-induced neuronal cell death and its underlying action mechanism(s) were investigated and compared to that of *α*-mangostin using primary cultured rat cortical cells. In addition, the effect of orally administered *γ*-mangostin on scopolamine-induced memory impairment was evaluated in mice. We found that *γ*-mangostin exhibited prominent protection against H_2_O_2_- or xanthine/xanthine oxidase-induced oxidative neuronal death and inhibited reactive oxygen species (ROS) generation triggered by these oxidative insults. In contrast, *α*-mangostin had no effects on the oxidative neuronal damage or associated ROS production. We also found that *γ*-mangostin, not *α*-mangostin, significantly inhibited H_2_O_2_-induced DNA fragmentation and activation of caspases 3 and 9, demonstrating its antiapoptotic action. In addition, only *γ*-mangostin was found to effectively inhibit lipid peroxidation and DPPH radical formation, while both mangostins inhibited *β*-secretase activity. Furthermore, we observed that the oral administration of *γ*-mangostin at dosages of 10 and 30 mg/kg markedly improved scopolamine-induced memory impairment in mice. Collectively, these results provide both *in vitro* and *in vivo* evidences for the neuroprotective and memory enhancing effects of *γ*-mangostin. Multiple mechanisms underlying this neuroprotective action were suggested in this study. Based on our findings, *γ*-mangostin could serve as a potentially preferable candidate over *α*-mangostin in combatting oxidative stress-associated neurodegenerative diseases including Alzheimer's disease.

## 1. Introduction

The generation of reactive oxygen species (ROS) including superoxide anion (·O_2_^−^), hydroxyl radical (·OH), and hydrogen peroxide is recognised as a key factor in oxidative stress to the cells [1]. The accumulation of ROS in neuronal cells causes lipid peroxidation as well as damage to the structure and function of proteins and DNA molecules, ultimately leading to cell death [2]. The brain is known to be particularly susceptible to oxidative stress, due to its relative deficiency of endogenous antioxidant defence mechanisms, enriched levels of transition metals and unsaturated lipids, and high utilisation of oxygen [3]. Consequently, oxidative stress-induced neuronal damage has been recognised as an important mechanism involved in many neurodegenerative diseases, including Alzheimer's disease (AD) and Parkinson's disease [4, 5].

AD is characterised by the progressive impairment of cognition which is strongly correlated with neuronal degeneration and death. One of the hallmarks of AD is the appearance of senile plaques generated through the extracellular deposition of *β*-amyloid (A*_β_*) peptide, which is derived from amyloid precursor protein (APP) upon enzymatic cleavage by *β*- and *γ*-secretases [6, 7]. A*_β_* peptide can induce oxidative damage through the production of ROS, potentially triggering neurotoxic events. Furthermore, it has been suggested that an augmentation in the cellular level of ROS and free radicals produces more A*_β_* peptide, which in turn exerts further oxidative stress and toxic insults on neurons [8]. With the accumulative evidence for oxidative stress as an important factor in AD, antioxidants that reduce ROS and prevent oxidative stress-induced neuronal death have been intriguing potential candidates to prevent or treat AD [4, 5]. However, the results from many clinical studies have been rather disappointing so far. Nonetheless, various approaches considering heterogeneous and multifactorial characteristics of AD are attempted to explore favorable efficacy of antioxidant therapy in AD [9].

Mangosteen, *Garcinia mangostana* L. (*G. mangostana,* Guttifereae), is a tree cultivated in Southeast Asia including Indonesia, Philippines, and India. Its fruit is edible and also known to have medicinal benefits. The pericarp of the fruit has been traditionally used in these countries to treat infection, wounds, inflammation, and diarrhea [10]. In addition, mangosteen products in the form of juice or tablets account for some of the best-selling dietary supplements in the U.S. market [11]. The major bioactive secondary metabolites of mangosteen have been found to be xanthone derivatives, among which *α*-mangostin is the most studied xanthone [12, 13]. Using cell-free *in vitro* assays, *α*-mangostin was reported to scavenge singlet oxygen and superoxide anion, while it was shown to be unable to scavenge hydroxyl radicals and hydrogen peroxide [14]. Additionally, using primary cultures of cerebellar granule neurons, *α*-mangostin was found to exhibit ROS scavenging and neuroprotective effects against the mitochondrial toxin 3-nitropropionic acid or iodoacetate, an inhibitor of the glycolytic enzyme glyceraldehyde-3-phosphate dehydrogenase inducing metabolic inhibition in neurons [14, 15]. Moreover, *α*-mangostin has been demonstrated to attenuate *β*-amyloid oligomers-induced neurotoxicity by inhibiting amyloid aggregation and also to decrease A*_β_* production via modulation of the amyloidogenic pathway [16, 17]. Together, these findings suggest that *α*-mangostin may serve as a multifunctional therapeutic intervention to combat the multiple pathological processes of AD [18, 19].

Unlike *α*-mangostin, however, there have only been limited findings supporting the neuroprotective effects of *γ*-mangostin. It has been reported that *γ*-mangostin, along with other xanthones from *G. mangostana*, inhibits glutamate-induced cell death in the HT22 hippocampal neuronal cell line and self-induced A_*β*42_ aggregation *in vitro* [18]. These findings also suggest that, in addition to *α*-mangostin, *γ*-mangostin could be a promising compound for AD therapy [18].

In order to confirm and further characterise the neuroprotective actions of these mangostins, the present study evaluated the effect of *γ*-mangostin on the oxidative insults using primary cultured rat cortical cells as a model and compared it to that of *α*-mangostin. To elucidate the probable action mechanism(s) underlying the neuroprotective effect, we next investigated the effects on the H_2_O_2_-induced DNA fragmentation and activation of caspases. Their antioxidant properties and effects on *β*-secretase activity were further examined using cell-free *in vitro* assays. In order to provide *in vivo* evidence of its therapeutic potential in AD, we finally evaluated the memory enhancing effect of orally administered *γ*-mangostin in a mouse model of scopolamine-induced memory impairment using the passive avoidance test.

## 2. Materials and Methods

### 2.1. Materials

**Figure 1:**
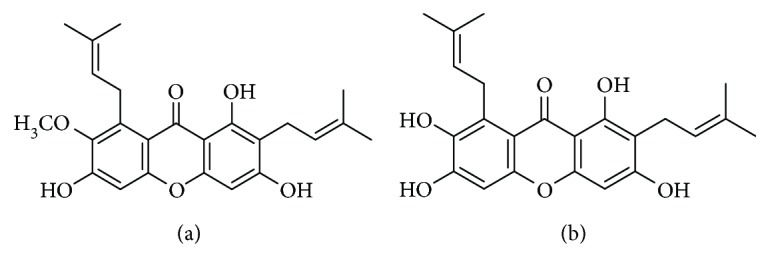
Chemical structures of *α*-mangostin (a) and *γ*-mangostin (b) isolated from *G. mangostana*.

*G. mangostana* fruits were collected from Indonesia, and their pericarps were extracted with methanol. Further separation and identification of *α*- and *γ*-mangostins were performed according to the method described previously [20]. Spectroscopic data for *α*- and *γ*-mangostins are depicted in Supplementary Materials (available here), and their chemical structures are shown in Figure 1. The purification yields of *α*- and *γ*-mangostins were 0.57% (*w*/*w*) and 0.06% (*w*/*w*), respectively, based on the total weight of mangosteen pericarp. The purity of these compounds as determined by HPLC-UV analysis was >95%. Minimum essential medium (MEM, supplemented with Earle's salt), fetal bovine serum (FBS), horse serum (HS), and antibiotic-antimycotic agent were procured from Invitrogen (Carlsbad, CA, USA). Laminin, poly-L-lysine, glucose, L-glutamine, 3-(4,5-dimethylthiazol-2-yl)-2,5-diphenyltetrazolium bromide (MTT), 2′,7′-dichlorofluorescin diacetate (DCFH-DA), H_2_O_2_, xanthine (X), xanthine oxidase (XO), cytosine arabinoside, 2-thiobarbituric acid (TBA), 1,1-diphenyl-2-picrylhydrazyl (DPPH), and anti-*β*-actin antibody (monoclonal) were obtained from Sigma-Aldrich (St. Louis, MO, USA). Polyethylene glycol (PEG) was from Yakuri Pure Chemicals Co. Ltd. (Kyoto, Japan). Anti-caspase 3 (8G10), anti-caspase 9 (Asp353), and horseradish peroxidase- (HRP-) linked anti-rabbit or anti-mouse immunoglobulin G (IgG) antibodies were purchased from Cell Signaling Technology (Danvers, MA, USA).

### 2.2. Animals

Timed-pregnant Sprague-Dawley (SD) rats and ICR mice were procured from Daehan Biolink (Chungbuk, Korea). Animals were maintained under conditions of controlled temperature (22 ± 2°C) and relative humidity (40-60%) with a 12 h light-dark cycle. They were given access to a standard chow diet and water *ad libitum*. All experimental procedures including the use, care, and handling of animals were conducted following the international guidelines (Guide for the Care and Use of Laboratory Animals, Institute of Laboratory Animal Resources, Commission on Life Sciences, National Research Council; National Academy Press: Washington D.C., 1996). Prior to the study, the rationale, design, and protocols of the experiments were approved by the Institutional Animal Ethical Committee of Dongguk University (approval numbers: IACUC-2013-0005 and IACUC-2016-035-2).

### 2.3. Cell Culture

Primary culture of rat cerebrocortical cells containing neuronal and nonneuronal cells was carried out as previously described [21, 22]. Briefly, pregnant SD rats on the 17th day of gestation were sacrificed using anesthesia, and their uteri were promptly removed. Embryos were harvested, and their cerebral cortices were excised and mechanically dissociated into single cells by triturating with fire-polished Pasteur pipettes. The isolated cells were then seeded on either 35 mm culture dishes (6 × 10^6^ cells/dish) or 24-well culture plates (6 × 10^5^ cells/well) precoated with the mixture of poly-L-lysine and laminin in MEM (containing Earle's salt) supplemented with 2 mM glutamine, 25 mM glucose, 5% FBS, 5% HS, and 1% antibiotic-antimycotic agent. The cultures plated on 35 mm dishes were employed for Western blotting analysis, and those on 24-well plates were for the remaining experiments. The cells were maintained in the same medium in an incubator at 37°C with a humidified atmosphere of 95% air/5% CO_2_. On day 7 of plating, the cultures were treated with 10 *μ*M cytosine arabinoside in order to arrest the proliferation of nonneuronal cells. Finally, the neuronal cells were used for experimentation on days 10-11 of culturing.

### 2.4. Treatment of Cells and Assessment of Cell Viability

Before starting any treatment, the cultured cortical cells were washed with HEPES-buffered control salt solution (HCSS, 20 mM HEPES, pH 7.4; 120 mM NaCl; 5.4 mM KCl; 1.6 mM MgCl_2_·6H_2_O; 2.3 mM CaCl_2_·2H_2_O; 15 mM glucose; 10 mM NaOH). To assess the potential cytotoxic effects of *α*- or *γ*-mangostin, the HCSS-washed cultured cells were treated with these compounds at the concentrations of 0.3~10 *μ*M in MEM supplemented with 25 mM glucose (MEMG) for 24 h. To induce oxidative damage, the cultured cells were exposed to 100 *μ*M H_2_O_2_ for 5 min or 0.5 mM X and 10 mU/ml XO for 10 min in MEMG, washed with HCSS, and then incubated in MEMG for 18-20 h [23]. For each experiment, the control cells were exposed to the vehicle (MEMG) without any agent.

In order to evaluate the protective effects of *α*- or *γ*-mangostin on the oxidative neuronal damage elicited by the above-mentioned inducers, the cultured cells were simultaneously treated with mangostin compounds at the concentrations of 0.3~10 *μ*M with the respective insults.

Following the termination of desired treatments, the viability of cells was determined by the MTT reduction assay, as previously described [22, 23]. In brief, MTT was added to the treated cells at a final concentration of 1.0 mg/ml and incubated for 3 h at 37°C. Upon completion of the MTT reaction, the culture media were carefully removed, and 500 *μ*l DMSO was added. Following the incubation of cells for 15 min to dissolve the formazan crystal products, the absorbance was measured at 550 nm using a microplate reader (SpectraMax M2^e^, Molecular Devices, Sunnyvale, CA, USA). The viability of control cells in terms of absorbance was expressed as 100%.

### 2.5. Determination of Intracellular ROS

The effect of *α*- or *γ*-mangostin on the generation of intracellular ROS was measured spectrofluorometrically using DCFH-DA fluorogenic dye as a probe [22]. Briefly, after washing with HCSS, the cultured cells were treated with DCFH-DA at a final concentration of 10 *μ*M in MEMG for 30 min at 37°C, washed with HCSS, and then treated with the corresponding insults (100 *μ*M H_2_O_2_ or 0.5 mM X in combination with 10 mU/ml XO) in MEMG for 2 h in the absence or presence of *α*- or *γ*-mangostin at the concentrations of 0.3~10 *μ*M. Intracellular ROS generation was determined by the fluorescence detection of 2′,7′-dichlorofluorescein on a microplate reader (SpectraMax M2^e^, Molecular Devices) with excitation and emission wavelengths at 490 nm and 520 nm, respectively, and expressed as % control.

### 2.6. Fluorescence Microscopy

The effect of *α*- or *γ*-mangostin on the H_2_O_2_-induced ROS generation in the cultured cells was further confirmed by fluorescence microscopy using DCFH-DA fluorogenic dye as a probe. Briefly, following DCFH-DA probing and subsequent treatment with 100 *μ*M H_2_O_2_ in the absence or presence of either *α*- or *γ*-mangostin (10 *μ*M) as described above, the intracellular ROS was visualised under a Nikon Eclipse Ti-U inverted microscope (Nikon, Tokyo, Japan) with excitation and emission wavelengths of 495 and 530 nm, respectively [24].

### 2.7. Terminal Deoxynucleotidyl Transferase-Mediated Deoxyuridine Triphosphate (dUTP) Nick End-Labeling (TUNEL) Assay

The effect of *α*- or *γ*-mangostin at 10 *μ*M on H_2_O_2_-treated apoptotic cells was evaluated by detection of fragmented DNA using a TUNEL assay kit (DeadEnd™ Colorimetric TUNEL System, Promega, Madison, WI, USA), performed according to the manufacturer's instructions [22]. Briefly, the cells were treated, washed with PBS, and fixed for 25 min in 4% paraformaldehyde. The cells were then washed with PBS and incubated for 5 min with 0.2% Triton X-100 for permeabilisation. Following washing and incubation in equilibration buffer for 10 min at room temperature, the cells were transferred into terminal deoxynucleotidyl transferase reaction mixture containing biotinylated nucleotide mix and then incubated for 60 min at 37°C to permit the nick end-labeling reaction. After terminating the reaction, the cells were immersed in saline sodium citrate solution and subsequently incubated with streptavidin-conjugated HRP. After washing with PBS, the cells were finally stained with diaminobenzidine. The cells were washed twice with PBS, and the TUNEL-positive cells stained as dark brown color were detected using a TS-100 inverted microscope (Nikon, Tokyo, Japan).

### 2.8. Western Blotting

The immunodetection of the cleaved caspases 3 and 9 was performed by Western blotting as previously described [25]. Briefly, the cultured cells were serum-starved overnight, exposed to either *α*- or *γ*-mangostin for 30 min in serum-free medium prior to cotreatment with 100 *μ*M H_2_O_2_ for 2 h, and then the cells were lysed for 30 min on ice in the lysis buffer (10 mM Tris-HCl, pH 7.4; 150 mM NaCl; 2 mM EDTA; 4.5 mM sodium pyrophosphate; 10 mM *β*-glycerophosphate; 1 mM NaF; 1 mM Na_3_VO_4_; 1% (*v*/*v*) Triton X-100; 0.5% (*v*/*v*) NP-40; and one tablet of protease inhibitor cocktail (Roche Diagnostic GmbH, Mannheim, Germany)). The resultant lysates were then centrifuged at 14,000 rpm for 30 min at 4°C, and the supernatants were collected. The protein concentrations of the supernatants were determined using a Bio-Rad D_C_ protein assay kit (Bio-Rad, Hercules, CA, USA). Equal amounts of lysate proteins (30 *μ*g) were then resolved by sodium dodecyl sulfate-polyacrylamide gel electrophoresis on 12% gels and electrophoretically transferred to nitrocellulose membranes (Whatman, Clifton, NJ, USA) for 1.5 h at 100 V. After blocking for 1.5 h with Tris-buffered saline (TBS) containing 0.1% Tween 20 (TBST) and 5% nonfat dry milk (BD Falcon, Sparks, MD, USA), the membranes were incubated overnight at 4°C with anti-caspase 3 or 9 antibodies in TBST containing 5% bovine serum albumin (USB, Canton, OH, USA). Next, the membranes were washed three times with TBST and incubated for 1.5 h with HRP-conjugated anti-rabbit IgG secondary antibody. The immunoreactive bands in the membranes were detected by a Bio-Rad ChemiDoc XRS imaging system (Bio-Rad) using Super Signal West Pico ECL reagent (Thermo Fisher Scientific, San Jose, CA, USA). In order to detect *β*-actin as an internal control, the membranes were stripped and incubated with anti-*β*-actin antibody.

### 2.9. Determination of DPPH Radical Scavenging Activity

The effect of *α*- or *γ*-mangostin on DPPH radicals was measured as previously described [26]. Briefly, the reaction mixture containing *α*- or *γ*-mangostin at the concentrations of 0.3~30 *μ*M and methanolic solution of DPPH (150 *μ*M) was incubated at 37°C for 30 min. The absorbance was then measured at 520 nm on a microplate reader (SpectraMax M2^e^, Molecular Devices). The radical scavenging activity of the samples was determined as % inhibition of DPPH absorbance using the following equation:

(1) $$\begin{array}{c} \text{Inhibition}\left(\%\right)=100\times \frac{\left({\text{Abs}}_{\text{control}}-{\text{Abs}}_{\text{sample}}\right)}{{\text{Abs}}_{\text{control}}}, \end{array}$$

where Abs_control_ represents the absorbance of the control (without test sample) and Abs_sample_ denotes the absorbance in the presence of the test sample.

### 2.10. Assay of Lipid Peroxidation (LPO) in Rat Brain Homogenates

The effect of *α*- or *γ*-mangostin on LPO initiated by Fe^2+^ (10 *μ*M) and L-ascorbic acid (100 *μ*M) in the rat forebrain homogenates was measured as previously described [26]. Briefly, the reaction mixture was incubated at 37°C for 1 h in the absence (control) or presence of *α*- or *γ*-mangostin at the concentrations of 0.3~30 *μ*M. After stopping the reaction by adding trichloroacetic acid (28%*w*/*v*) and TBA (1%*w*/*v*), the mixture was heated at 100°C for 15 min and centrifuged to remove the precipitates. The absorbance of the supernatant was read at 532 nm on a microplate reader (SpectraMax M2^e^, Molecular Devices), and the percent inhibition of LPO was calculated using the above formula.

### 2.11. Assay of *In Vitro β*-Secretase Activity

The effect of *α*- or *γ*-mangostin on the *β*-secretase activity was determined using a *β*-secretase fluorescence resonance energy transfer (FRET) assay kit (Invitrogen, Carlsbad, CA, USA) following the manufacturer's instructions with some modifications [22]. In brief, a 10 *μ*l aliquot of assay buffer containing *α*- or *γ*-mangostin at the concentrations of 0.3~10 *μ*M was mixed with 20 *μ*l of the substrate (750 nM) in a 96-well plate. Subsequently, 10 *μ*l of *β*-secretase enzyme (1.0 U/ml) was added and incubated for 2 h at room temperature. The fluorescence was measured on a microplate reader (SpectraMax M2^e^, Molecular Devices) with excitation and emission wavelengths set at 545 and 585 nm, respectively.

### 2.12. Passive Avoidance Test

The passive avoidance test was performed as previously described [27, 28] on six-week-old ICR mice (28~30 g body weight) using two identical compartments (lighted and dark compartments) with an automated guillotine door in between them (Gemini Avoidance System, San Diego Instruments Inc., San Diego, CA, USA). For the acquisition trial, the animals were placed in the lighted chamber, and the guillotine door was opened 15 s later. After the animals entered into the dark compartment, the door was automatically shut down, and an electrical foot shock (0.5 mA for 5 s) was delivered to the animals through the grid floor. Twenty-four hours after the acquisition trial, the animals were again placed in the lighted compartment in order to conduct a retention trial. The duration of each trial was 300 s, and the time latency for entry into the dark compartment was measured.

The animals were orally administered with *γ*-mangostin (5, 10, and 30 mg/kg in 40%*v*/*v* PEG in water), donepezil (10 mg/kg), or vehicle (for control and scopolamine groups). After 30 min of administration, memory impairment was induced by intraperitoneal administration of scopolamine (3 mg/kg, prepared in normal saline); the control group received normal saline only. Following 30 min of scopolamine injection, the acquisition trial was initiated as described above.

### 2.13. Statistical Analysis

All experiments were performed individually at least three times. Quantitative data are expressed as the mean ± S.E.M. Statistical analyses were performed by one-way ANOVA followed by Tukey's post hoc test using SigmaPlot 12.5 software (Systat Software, San Jose, CA, USA). A *P* < 0.05 was considered to be significant.

## 3. Results

### 3.1. Effects of *α*- and *γ*-Mangostins on Neuronal Cell Viability in Primary Cultured Rat Cortical Cells

**Figure 2:**
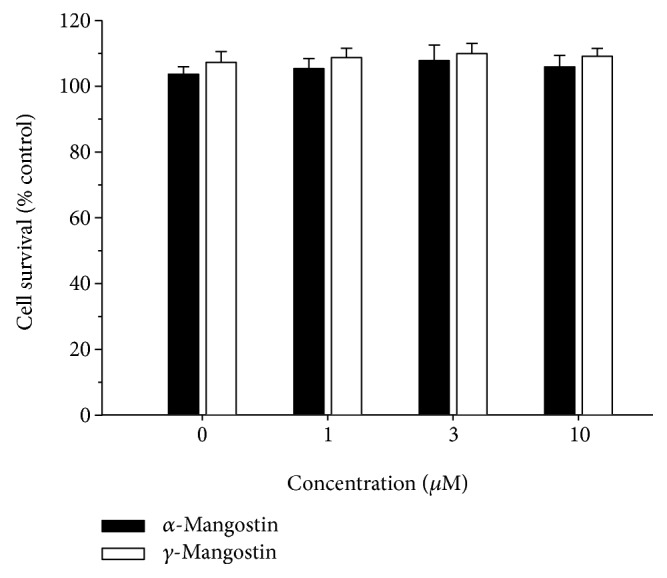
Effects of *α*- and *γ*-mangostins on neuronal cell viability in primary cultured rat cortical cells. Cells were exposed to the indicated concentrations of *α*- or *γ*-mangostin for 24 h. The control cells were treated with vehicle (DMSO) only. Cell viability was determined by the MTT reduction assay, as described in Materials and Methods. The viability of control cells treated with vehicle only was considered to be 100%, and the data were expressed as percentages of the control. Each point represents the mean ± S.E.M. from at least three independent experiments, performed in duplicate.

Exposure of the primary cultured rat brain cortical cells to either *α*- or *γ*-mangostin at concentrations ranging from 0.3~10 *μ*M for 24 h did not produce any cytotoxicity (Figure 2). Accordingly, upcoming experiments with *α*- or *γ*-mangostin were performed using this concentration range.

### 3.2. Effects of *α*- and *γ*-Mangostins on the H_2_O_2_- or X/XO-Induced Oxidative Neuronal Damage and ROS Generation in Primary Cultured Rat Cortical Cells

**Figure 3:**
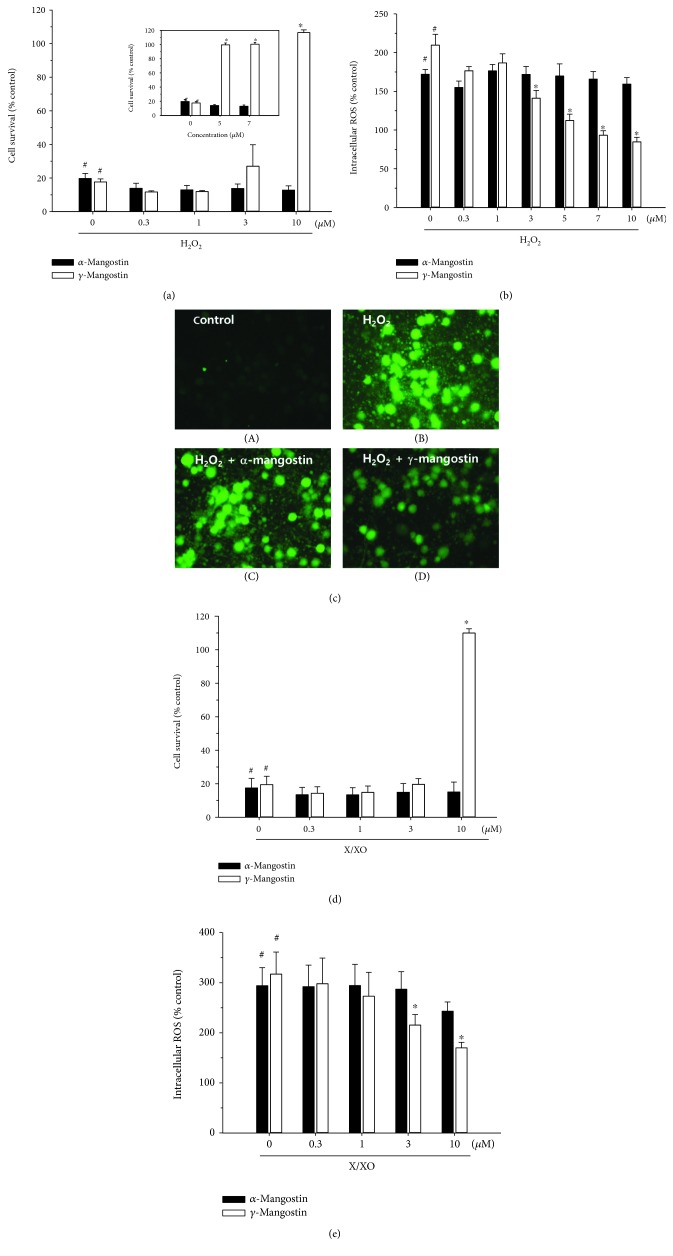
Effects of *α*- and *γ*-mangostins on H_2_O_2_- or X/XO-induced oxidative neurotoxicity and ROS generation in primary cultured rat cortical cells. (a and d) The cells were exposed to 100 *μ*M H_2_O_2_ for 5 min (a) or 0.5 mM X in combination with 10 mU/ml XO for 10 min (d) in the absence or presence of either *α*- or *γ*-mangostin at various concentrations as indicated. Cell viability was determined by the MTT reduction assay at 18-20 h after exposure, as described in Materials and Methods. The cell survival was expressed as percentages of the control treated with vehicle only. (b and e) The cells were preincubated with 10 *μ*M DCFH-DA for 30 min at 37°C in the dark, then treated with 100 *μ*M H_2_O_2_ for 2 h (b) or 0.5 mM X in combination with 10 mU/ml XO for 2 h (e) in the absence or presence of either *α*- or *γ*-mangostin at various concentrations as indicated. The generation of intracellular ROS was measured as described in Materials and Methods. The ROS levels were expressed as percentages of the control treated with vehicle only. Each data point represents the mean ± S.E.M. from at least three independent experiments, performed in duplicate (^#^ *P* < 0.05 vs. vehicle-treated control cells without *α*- or *γ*-mangostin treatment; ^∗^ *P* < 0.05 vs. H_2_O_2_- or X/XO-treated cells). (c) Fluorescence microscopic images showing the inhibition of H_2_O_2_-induced ROS generation by *γ*-mangostin in primary cultured rat cortical cells. The cells were preincubated with 10 *μ*M DCFH-DA for 30 min at 37°C in the dark and treated with 100 *μ*M H_2_O_2_ in the absence (B) or presence of 10 *μ*M *α*-mangostin (C) or *γ*-mangostin (D) for 2 h. The control cells were treated with vehicle only without *α*- or *γ*-mangostin (A). Following the desired treatment, ROS levels were imaged using epifluorescence microscopy as described in Materials and Methods. Representative photomicrographs from three independent experiments are shown.

In agreement with the previous reports [22, 26], treatment of the cultured cells with 100 *μ*M of H_2_O_2_ for 5 min caused approximately 80% or more cell death and approximately 200% increase in intracellular ROS production (Figures 3(a) and 3(b), respectively (#, *P* < 0.05 vs. vehicle-treated control cells without H_2_O_2_, *α*-mangostin, or *γ*-mangostin treatment)). The reduced viability of the H_2_O_2_-treated cells was completely reversed by *γ*-mangostin at the concentration of 10 *μ*M (Figure 3(a) (^∗^, *P* < 0.05 vs. H_2_O_2_-treated cells without *α*- or *γ*-mangostin)). Since its protective effect against the H_2_O_2_-induced oxidative damage was so dramatic, we further tested the effect of *γ*-mangostin at the concentrations between 3 and 10 *μ*M. As shown in Figure 3(a) (inset), 5 and 7 *μ*M of *γ*-mangostin also exhibited dramatic increases in the viability of H_2_O_2_-treated cells. In addition, the H_2_O_2_-induced ROS production was significantly inhibited by *γ*-mangostin at 3~10 *μ*M concentrations (Figure 3(b) (^∗^, *P* < 0.05 vs. H_2_O_2_-treated cells without *α*- or *γ*-mangostin)), which was confirmed by fluorescence microscopy (Figure 3(c)). In contrast, *α*-mangostin showed no significant effects on H_2_O_2_-induced oxidative damage or ROS production at the concentrations tested in this study (Figures 3(a)–3(c)).

Treatment of the cultured cells with 0.5 mM X and 10 mU/ml XO caused approximately 80% or more cell death and a 300% increase in ROS production (Figures 3(d) and 3(e), respectively (#, *P* < 0.05 vs. vehicle-treated control cells without X/XO, *α*-mangostin, or *γ*-mangostin treatment)). The decreased viability of the X/XO-treated cells was totally reversed by *γ*-mangostin at the concentration of 10 *μ*M (Figure 3(d) (^∗^, *P* < 0.05 vs. X/XO-treated cells without *α*- or *γ*-mangostin)). Similarly, the X/XO-induced ROS production was significantly suppressed by *γ*-mangostin at 3 and 10 *μ*M concentrations (Figure 3(e) (^∗^, *P* < 0.05 vs. X/XO-treated cells without *α*- or *γ*-mangostin)). However, *α*-mangostin showed no significant effects on X/XO-induced oxidative damage or ROS production at the concentrations tested (Figures 3(d) and 3(e)).

### 3.3. Effects of *α*- and *γ*-Mangostins on the H_2_O_2_-Induced Apoptosis in Primary Cultured Rat Cortical Cells

**Figure 4:**
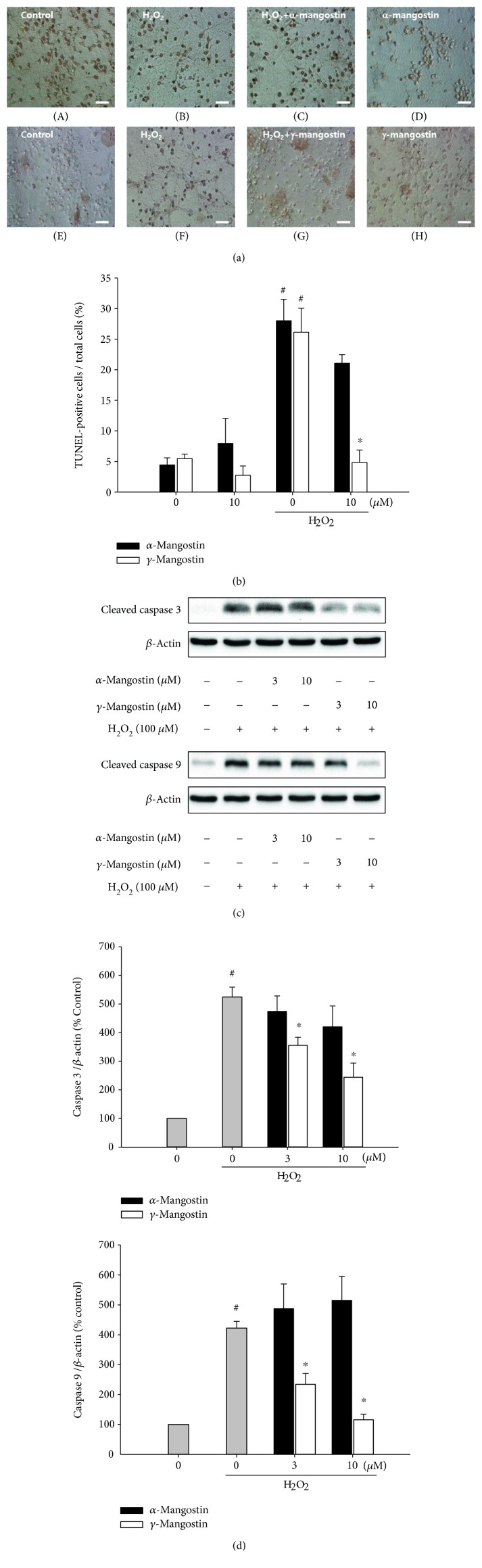
Effects of *α*- and *γ*-mangostins on H_2_O_2_-induced apoptosis in primary cultured rat cortical cells. (a and b) Inhibition of H_2_O_2_-induced DNA fragmentation by *γ*-mangostin. Cells were treated with 100 *μ*M H_2_O_2_ for 2 h with or without *α*- or *γ*-mangostin at the concentration of 10 *μ*M, and the TUNEL assay was carried out as described in Materials and Methods. Representative microscopic images from at least three individual experiments are shown (a). (A and E) Control cells were treated with vehicle only; (B and F) cells were treated with 100 *μ*M H_2_O_2_ for 2 h; (C and G) cells were treated for 2 h with either 10 *μ*M *α*-mangostin (C) or *γ*-mangostin (G) in combination with 100 *μ*M H_2_O_2_; (D and H) cells were treated with 10 *μ*M *α*-mangostin (D) or *γ*-mangostin for 2 h without H_2_O_2_ (H). Scale bar = 10 *μ*m. Quantitative analyses of the TUNEL-positive cells from at least three independent experiments are shown (b) (^#^ *P* < 0.05 vs. vehicle-treated control cells without *α*- or *γ*-mangostin treatment; ^∗^ *P* < 0.05 vs. H_2_O_2_-treated cells without *α*- or *γ*-mangostin). (c and d) Inhibition of the H_2_O_2_-induced activation of caspases 3 and 9 by *γ*-mangostin. Cells were treated with 100 *μ*M H_2_O_2_ for 2 h in the absence or presence of either *α*- or *γ*-mangostin at 3 and 10 *μ*M. The expression of cleaved caspases 3 and 9 was assessed by Western blotting as described in Materials and Methods. Representative blots from at least three individual experiments are shown (c). The intensities of the bands from at least three independent experiments were quantified by densitometric analyses and normalised to *β*-actin (d) (^#^ *P* < 0.05 vs. vehicle-treated control cells without *α*- or *γ*-mangostin treatment; ^∗^ *P* < 0.05 vs. H_2_O_2_-treated cells without *α*- or *γ*-mangostin).

In order to elucidate the probable mechanism(s) underlying the neuroprotective role of *γ*-mangostin, we next examined the effect of this compound on H_2_O_2_-induced apoptosis and compared it to that of *α*-mangostin. In agreement with the previous report [29], the exposure of cultured cells to H_2_O_2_ caused DNA fragmentation, an important hallmark of apoptosis, as reflected by a dramatic increase in the TUNEL-positive cell population (Figures 4(a) (B and F) and Figure 4(b) (#, *P* < 0.05 vs. vehicle-treated control cells without *α*- or *γ*-mangostin treatment)). The H_2_O_2_-induced DNA fragmentation was remarkably inhibited by *γ*-mangostin at 10 *μ*M (Figure 4(a) (G) and Figure 4(b) (^∗^, *P* < 0.05 vs. H_2_O_2_-treated cells without *α*- or *γ*-mangostin treatment)). We also examined their effects on the H_2_O_2_-induced activation of caspases, another important molecular event during the apoptotic process. As illustrated in Figures 4(c) and 4(d), *γ*-mangostin significantly attenuated the H_2_O_2_-induced activation of both caspases 3 and 9. In contrast, *α*-mangostin neither prevented DNA fragmentation nor inhibited caspase activities in the H_2_O_2_-treated cells (Figure 4).

### 3.4. Effects of *α*- and *γ*-Mangostins on DPPH Radical Formation and Lipid Peroxidation

**Figure 5:**
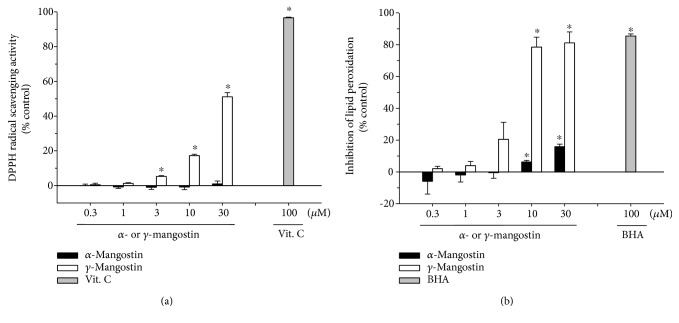
Effects of *α*- and *γ*-mangostins on DPPH radical formation and lipid peroxidation (LPO). Inhibition of DPPH radical (a) and LPO induced by Fe^2+^ (10 *μ*M) and L-ascorbic acid (100 *μ*M) in rat forebrain homogenate (b) by *α*- or *γ*-mangostin at the indicated concentrations were measured as described in Materials and Methods. Each data point represents the mean ± S.E.M. from at least three independent experiments, performed in duplicate (^∗^ *P* < 0.05 vs. vehicle-treated control without *α*- or *γ*-mangostin treatment). Vit. C and BHA were used as references to validate the assay procedures for DPPH radical scavenging activity and inhibition of LPO, respectively (grey bars). Vit. C: vitamin C; BHA: butylhydroxyanisole.

The antioxidant properties of *α*- and *γ*-mangostins were further substantiated by evaluating their radical scavenging activities using stable free radical DPPH as a probe (Figure 5(a)). In addition, their ability to inhibit LPO initiated by Fe^2+^ and L-ascorbic acid in rat brain homogenate was also examined (Figure 5(b)). Our results demonstrated that *γ*-mangostin considerably attenuated the formation of DPPH radicals and effectively inhibited lipid peroxide formation in concentration-dependent manners. In contrast, *α*-mangostin showed no DPPH radical scavenging activity with only a minimal inhibition of LPO at the concentrations tested (Figures 5(a) and 5(b), respectively). Vit. C and BHA were used as reference compounds to validate the assay procedures for DPPH radical scavenging activity and inhibition of LPO, respectively (grey bars).

### 3.5. Effects of *α*- and *γ*-Mangostins on *In Vitro β*-Secretase Enzyme Activity

**Figure 6:**
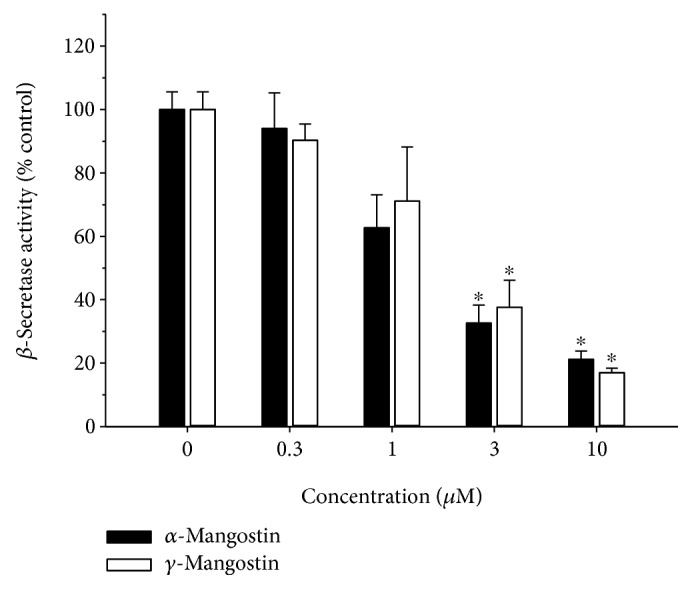
Effects of *α*- and *γ*-mangostins on *β*-secretase activity. The inhibitory effects of *α*- and *γ*-mangostins on the enzymatic activity of *β*-secretase were determined by the *β*-secretase FRET assay as described in Materials and Methods. The data were expressed as percentages of the control treated without *α*- or *γ*-mangostin. Each data point represents the mean ± S.E.M. from at least three independent experiments, performed in duplicate (^∗^ *P* < 0.05 vs. vehicle-treated control without *α*- or *γ*-mangostin treatment).

Since *β*-secretase plays a vital role in the generation of the A*_β_* peptide from APP [6, 7], we also examined the impact of *α*- and *γ*-mangostins on the activity of this enzyme using the *in vitro β*-secretase FRET assay kit. We found that both *α*- and *γ*-mangostins potently inhibited *β*-secretase activity in concentration-dependent fashion (Figure 6).

### 3.6. Effect of *γ*-Mangostin on the Scopolamine-Induced Memory Impairment in Mice

Using cell-based and cell-free *in vitro* assays, our findings demonstrated that *γ*-mangostin showed more potent antioxidant and neuroprotective activities than *α*-mangostin, although both compounds showed similar degrees of inhibition against *β*-secretase activity. Based on these findings, we selected *γ*-mangostin to examine whether it could improve scopolamine-induced memory impairment in mice using the passive avoidance test.

**Figure 7:**
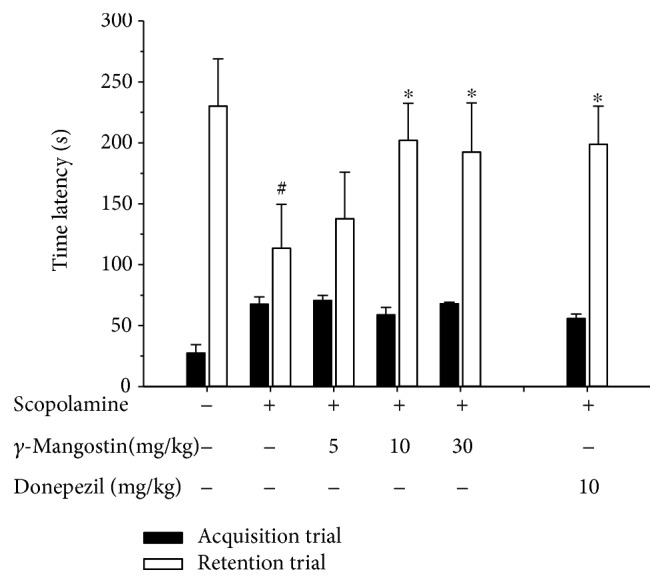
Effect of *γ*-mangostin on the scopolamine-induced memory impairment in mice. Animals were randomly divided into 6 groups with 6-7 mice in each group. To the 3 groups of animals, *γ*-mangostin was orally administered at the respective dosages of 5, 10, or 30 mg/kg as indicated. For the reference drug-treated group, donepezil was administered at the dosage of 10 mg/kg. For the control group (the vehicle-treated group without scopolamine, *γ*-mangostin, or donepezil treatment) and the scopolamine group (the group treated with scopolamine injection, not with *γ*-mangostin or donepezil treatment), vehicle was only administered. After 30 min of each administration, memory impairment was induced by intraperitoneal injection of scopolamine (3 mg/kg in normal saline) in 5 groups as indicated above in the figure; for the control group, normal saline without scopolamine was injected. Following 30 min of scopolamine or saline injection, the acquisition trial was initiated by delivering a foot shock to the animals. Twenty-four hours after the acquisition trials, the retention trials were performed. The detailed experimental procedures are described in Materials and Methods. The time latency was calculated from three independent experiments. Each data point represents the mean ± S.E.M. (^#^ *P* < 0.05 vs. vehicle-treated control group without scopolamine, *γ*-mangostin, or donepezil treatment; ^∗^ *P* < 0.05 vs. scopolamine group treated with scopolamine only without *γ*-mangostin or donepezil).

As illustrated in Figure 7, the acquisition trials did not show significant differences in the time latency of all groups (black bars). During the retention trials (white bars), the scopolamine-treated group (without *γ*-mangostin or donepezil administration) showed marked reduction of the time latency than the control group treated with vehicle only (#, *P* < 0.05 vs. vehicle-treated control group), indicating that significant memory impairment was induced by scopolamine injection. The groups administered with *γ*-mangostin at the dosages of 10 and 30 mg/kg significantly restored the scopolamine-induced decrease in time latency (^∗^, *P* < 0.05 vs. scopolamine-treated amnesia group). Donepezil, a well-known cholinesterase inhibitor clinically used for the treatment of AD, was employed as a reference drug to validate our experimental procedures and compare its effect with that of *γ*-mangostin. The reduced time latency by scopolamine injection was also recovered by the oral administration of donepezil at the dosage of 10 mg/kg (^∗^, *P* < 0.05 vs. scopolamine-treated amnesia group). The inhibition of scopolamine-induced memory impairment by *γ*-mangostin administration (10 and 30 mg/kg of dosage) was nearly comparable to that of the donepezil-treated group (Figure 7). However, administration of *γ*-mangostin at the dosage of 5 mg/kg did not show a significant effect on reversing scopolamine-induced memory impairment in mice.

## 4. Discussion

Oxidative stress-induced cell damage is known to be involved in a number of neurodegenerative diseases such as AD, Parkinson's disease, and stroke [4, 5], where ROS such as superoxide anion, hydroxyl radical, and hydrogen peroxide play pivotal roles. It has been demonstrated that H_2_O_2_ can readily cross the cell membrane and cause injuries to the tissues through a number of different mechanisms, including the production of hydroxyl radicals and destabilisation of the oxidant/antioxidant pathway, ultimately leading to apoptotic and/or necrotic cell death [30]. The oxidation of X by XO also serves as an important source of ROS, generating H_2_O_2_ and superoxide anions that are known to contribute to the onset of neuronal damage in many neurodegenerative diseases [31, 32]. Taking critical roles of these ROS into account, we treated the primary cultured rat cortical cells with either H_2_O_2_ or X and XO in this study to induce oxidative neuronal damage as well as ROS generation and tested the effects of *α*- and *γ*-mangostins, the two xanthones isolated from the fruit hull of mangosteen, on this oxidative damage and ROS.

Our results demonstrated that the exposure of cultured cells to these oxidative insults produced approximately 80% cell death and marked increases in ROS production (Figures 3(a)–3(e)). We found in this study that only *γ*-mangostin, not *α*-mangostin, completely reversed the H_2_O_2_- or X/XO-induced oxidative neuronal damage and significantly attenuated ROS production. These results are in line with those of an earlier report, demonstrating that, among the sixteen xanthones including *α*- and *γ*-mangostins, only *γ*-mangostin exhibited HO^.^ scavenging activity in an *in vitro* cell-free assay [33].

It has been previously reported that *α*-mangostin provides ROS scavenging activity and neuroprotective action against 3-nitropropionic acid- or iodoacetic acid-treated primary cultures of cerebellar granule neurons [14, 15]. However, in our study, *α*-mangostin neither exhibited neuroprotective activity against the H_2_O_2_- or X/XO-induced oxidative damage nor inhibited associated ROS production at any concentration tested (Figures 3(a)–3(e)). The discrepancies between the earlier reports [14, 15] and our results may be due to the different cell types used in these studies. Another plausible reason for these contradictory results may be due to the different species of radicals generated by the different oxidative inducers used in these studies. According to the report by Pedraza-Chaverrí et al. [14], *α*-mangostin was able to scavenge superoxide anion and peroxynitrite anion, whereas it was unable to scavenge hydroxyl radicals and hydrogen peroxide. Since *α*-mangostin was found to inhibit 3-nitropropionic acid-induced neurotoxicity and ROS production, Pedraza-Chaverrí et al. suggested that superoxide radical and peroxynitrite anion may be involved in 3-nitropropionic acid-induced toxicity in cerebellar granule neurons [14]. In our study, however, hydroxyl radicals and hydrogen peroxide were produced in cortical neurons by the treatment with H_2_O_2_ and X/XO, and *α*-mangostin did not inhibit the neurotoxicity and ROS production caused by these inducers. Thus, our findings on *α*-mangostin are entirely in agreement with the observation by Pedraza-Chaverrí et al. It would be interesting to clarify preferable species of ROS, if any, to be scavenged by *γ*-mangostin under the same experimental conditions employed for *α*-mangostin. Further study will be required to elucidate underlying mechanisms by which these xanthones distinguish different species of radicals to scavenge. We further confirmed that *γ*-mangostin exhibits more potent radical scavenging activity and antioxidant effect than *α*-mangostin using *in vitro* assays for DPPH radical scavenging activity and LPO. Again, unlike *γ*-mangostin, *α*-mangostin showed no DPPH radical scavenging activity and only minimal inhibition of LPO (Figure 5).

The action of H_2_O_2_ as an inducer of apoptosis and DNA damage has been well documented [34, 35]. It has been found that in PC12 cells and SH-SY5Y neuroblatoma cells, H_2_O_2_ can induce ROS generation and the activation of caspase cascades and ultimately triggers apoptosis [29]. In agreement with these previous reports [29, 34, 35], the exposure of our cultured cells to H_2_O_2_ also caused DNA fragmentation (Figure 4(a), B and F), as evidenced by the increased number of TUNEL-positive cells (Figure 4(b)). The H_2_O_2_-induced DNA fragmentation was markedly inhibited by *γ*-mangostin but not by *α*-mangostin (Figures 4(a) and 4(b)). Furthermore, the H_2_O_2_ treatment of the cultured cells triggered the activation of caspases 3 and 9, the major molecular events in the apoptotic process. The activated caspases 3 and 9 were significantly suppressed only by *γ*-mangostin (Figures 4(c) and 4(d)). Recently, *γ*-mangostin was found to inhibit caspase 3 activity in 6-hydroxy dopamine-treated SH-SY5Y cells [36], which is consistent with our finding. Taken together, it is conceivable that the underlying mechanism(s) for the neuroprotective effect of *γ*-mangostin against H_2_O_2_-induced oxidative damage may involve the inhibition of ROS production, as well as its antiapoptotic action inhibiting DNA fragmentation and activation of caspases 3 and 9. Furthermore, the antioxidant activities of *γ*-mangostin, not *α*-mangostin, scavenging DPPH radical and inhibiting LPO may also contribute to its neuroprotective action (Figure 5).

Among the diverse pathophysiological factors involved in the onset and development of AD, A*_β_* peptide plays a key role in neuronal cell death [37]. In this study, we found that both *α*- and *γ*-mangostins inhibited *β*-secretase activity, an enzyme involved in the generation of the A*_β_* peptides from APP in the amyloidogenic pathway [6, 7]. Previously, a series of xanthones isolated from *G. mangostana* were reported to modestly inhibit BACE1 activity, exhibiting 60.3 and 42.1% inhibition by *α*- and *γ*-mangostins at the concentration of 100 *μ*M, respectively [17]. Our study expanded their findings, illustrating the concentration-dependent inhibition of *β*-secretase activity by both *α*- and *γ*-mangostins at the concentration range of 0.3~10 *μ*M (Figure 6). For some reason, however, the inhibition by *α*- or *γ*-mangostin was observed to be much more potent in our study than that shown by a previous report [18], as approximately 60-70% inhibition of *β*-secretase activity was achieved at the concentration as low as 3 *μ*M of *α*- or *γ*-mangostin (Figure 6). One of the possible explanations for this potency discrepancy may be due to the differences in the bioactivities of *β*-secretase in the assay kits employed in our study and in the previous report. In order to test this possibility, the *β*-secretase activity has to be reevaluated in the presence of the testing xanthones under the same experimental conditions, using assay kits manufactured by the same company. In any event, our and previous findings strongly support that both *α*- and *γ*-mangostins may reduce A*_β_* formation from APP through the inhibition of *β*-secretase activity.

It has been previously reported that *α*-mangostin concentration dependently attenuated the neurotoxicity induced by A_*β*-(1-40)_ or A_*β*-(1-42)_ oligomers in primary rat cerebral cortical neurons [16]. Based on molecular docking simulations, the thioflavin T fluorescence assay and electron microscopy imaging, *α*-mangostin was found to inhibit and dissociate amyloid aggregation, which could contribute to its effect of attenuating A*_β_* oligomers-induced neurotoxicity [16]. We also attempted to evaluate the effects of *α*- and *γ*-mangostins on the neurotoxicity induced in our culture model by A_*β*-(25-35)_, the active fragment of A*_β_*, as previously described [22]. We treated the cultured cells with 40 *μ*M of A_*β*-(25-35)_ for 24 h in the absence or presence of *α*- or *γ*-mangostin at the concentration range of 0.3~10 *μ*M. Both mangostins appeared to exhibit weak protective effect on A_*β*-(25-35)_-induced neurotoxicity (data not shown). However, the apparent effects were not statistically significant by one-way ANOVA analysis. Further extensive studies under the same experimental conditions with the same A*_β_* peptides may be necessary to evaluate and compare their effects on the A*_β_*-induced neurotoxicity.

It has long been believed that acetylcholine is one of the most important neurotransmitters in learning and memory processes [38], and thus, cholinergic dysfunction is closely associated with AD pathology [39, 40]. Based on this cholinergic hypothesis, AChE inhibitors such as donepezil are currently used to alleviate the symptoms of AD in clinical situations. Recently, several prenylated xanthones from mangosteen, including *α*- and *γ*-mangostins, have been reported to inhibit AChE activity with IC_50_ values of lower than 20.5 *μ*M, as determined by Ellman's colorimetric method [41, 42]. The protein-ligand interactions between AChE and xanthones were confirmed by molecular docking studies [41]. In our study, we also tested the effects of *α*- and *γ*-mangostins on AChE activity *in vitro* and verified the previous findings (data not shown). Collectively, based on our results and previous findings [18, 41, 42], both *α*- and *γ*-mangostins may reduce A*_β_* formation and improve cholinergic transmission through the inhibition of *β*-secretase and AChE activities, respectively.

The *α*- and *γ*-mangostins are the most extensively studied xanthone derivatives of mangosteen [12]. Although they share a common chemical backbone, their chemical structures differ with respect to the numbers of hydroxyl and methoxy groups (Figure 1). While *α*-mangostin possesses three hydroxyl groups (at positions 1, 3, and 6) and a methoxy group (at position 7), *γ*-mangostin has four hydroxyl groups (at positions 1, 3, 6, and 7) without any methoxy group. We observed that only *γ*-mangostin, not *α*-mangostin, exhibited marked and potent neuroprotective and antioxidant effects. The exact mechanisms by which the structural discrepancy between these two xanthones can account for such a decisive difference in their neuroprotective and antioxidant effects are not yet understood. The catechol moiety of *γ*-mangostin or the hydroxyl group itself at position 7 may play crucial role in its neuroprotective and radical scavenging activities. Interestingly, however, the structural difference between *α*- and *γ*-mangostins was not reflected in their inhibitory effects on *β*-secretase and AChE enzyme activities, as both mangostins exhibited similar degrees of inhibition (Figure 6) [41, 42]. It is assumed that the hydroxyl groups at positions 1, 3, and 6 may play important roles in the inhibition of these enzyme activities. The methoxy group at position 7 of *α*-mangostin may not be directly involved in the interactions with these enzymes. Further studies are needed to explain the basis of such differential or similar pharmacological actions by the two mangostin compounds.

Even though both mangostins exhibited similar inhibition against *β*-secretase and AChE activities as measured in cell-free *in vitro* assays, *γ*-mangostin appeared to be a substantially more potent antioxidant and neuroprotective agent than *α*-mangostin, based on our findings in cell-based as well as cell-free *in vitro* studies. These beneficial pharmacological profiles of *γ*-mangostin strongly suggest its therapeutic potential for AD and other neurodegenerative diseases associated with oxidative stress.

In order to test this possibility, we next conducted a passive avoidance test using a scopolamine-induced amnesia model in mice to investigate the memory-improving effect of *γ*-mangostin *in vivo*. It has been previously revealed that memory deficit induced by scopolamine, a nonselective muscarinic receptor antagonist, is associated with oxidative stress [43], an event known to play a vital role in neurodegenerative disorders such as AD [4, 5]. As shown in Figure 7, the memory impairment induced by scopolamine was significantly improved by a single oral administration of *γ*-mangostin at dosages of 10 and 30 mg/kg. The memory-improving effects of *γ*-mangostin at these dosages were quite comparable to that of donepezil, a reference drug. Our results are in alignment with those of a previous report, demonstrating the protective effect of the mangosteen extract on scopolamine-induced amnesia [28]. In addition, the ability of *γ*-mangostin to penetrate the blood-brain barrier and reach CNS targets was predicted *in vitro* using a parallel artificial membrane penetration assay [18]. Taken together, our result and the previous findings suggest that *γ*-mangostin could be a promising candidate for therapeutic interventions of AD. To the best of our knowledge, our present study is the first report revealing the neuroprotective effect of *γ*-mangostin against oxidative neurotoxicity, as well as its memory-enhancing effect in mice.

## 5. Conclusions

The present study demonstrated that *γ*-mangostin, a xanthone derivative isolated from the fruit hull of mangosteen, exhibited a potent neuroprotective effect against H_2_O_2_- or X/XO-induced oxidative neuronal damage. The underlying mechanisms for this neuroprotective action may involve the inhibition of ROS generation triggered by these oxidative insults, antioxidant activities as evident by inhibition of DPPH radical formation and LPO, and its antiapoptotic properties as demonstrated by the inhibition of H_2_O_2_-induced DNA fragmentation and activation of caspases. Unlike *γ*-mangostin, however, *α*-mangostin neither exhibited neuroprotective activity nor demonstrated antioxidant properties in our study. Moreover, *γ*-mangostin was shown to exhibit a potent inhibitory effect on *β*-secretase activity and strongly improved scopolamine-induced memory deficits in mice. Based on our study providing *in vitro* and *in vivo* evidences, *γ*-mangostin may be considered to be a promising candidate in the prevention and treatment of various neurodegenerative diseases including AD.

## Data Availability

The data used to support the findings of this study are available from the corresponding author upon request.

## Abbreviations

A*_β_*: *β*-Amyloid
AD: Alzheimer's disease
APP: Amyloid precursor protein
DCFH-DA: 2′,7′-Dichlorodihydrofluorescein diacetate
DMSO: Dimethylsulfoxide
DPPH: 1,1-Diphenyl-2-picrylhydrazyl
FBS: Fetal bovine serum
FRET: Fluorescence resonance energy transfer
*G. mangostana*: *Garcinia mangostana* L.
HCSS: HEPES-buffered control salt solution
HRP: Horseradish peroxidase
HS: Horse serum
IgG: Immunoglobulin G
MEM: Minimum essential medium
MEMG: MEM supplemented with 25 mM glucose
MTT: 3-(4,5-Dimethylthiazol-2-yl)-2,5-diphenyltetrazolium bromide
ROS: Reactive oxygen species
SD: Sprague-Dawley
TBA: 2-Thiobarbituric acid
TBS: Tris-buffered saline
TBST: Tris-buffered saline containing 0.1% Tween 20
TUNEL: Terminal deoxynucleotidyl transferase-mediated deoxyuridine triphosphate nick end-labeling
X: Xanthine
XO: Xanthine oxidase.
