# Supplementary material for: Inhibition of Oxidative Neurotoxicity and Scopolamine-Induced Memory Impairment by γ-Mangostin: In Vitro and In Vivo Evidence
Source: Oxid Med Cell Longev. 2019 Mar 24;2019:3640753. doi: 10.1155/2019/3640753 (PMC6451816; doi:10.1155/2019/3640753)
Supplement: Supplementary Materials — Spectroscopic data are available as Supplementary Materials. [file 3640753.f1.pdf]

Supplementary information

Inhibition of oxidative neurotoxicity and scopolamine-induced memory impairment by γ-mangostin: *In vitro* and *in vivo* evidence

Youngmun Lee[#], Sunyoung Kim[#], Yeonsoo Oh, Young-Mi Kim, Young-Won Chin and Jungsook Cho*

College of Pharmacy, Dongguk University-Seoul, Goyang, Gyeonggi-do 10326, Republic of Korea

[#]These two authors contributed equally.

There are six figures depicting HPLC, ^{1}H-NMR, ^{13}C-NMR data of α- and γ-mangostins, respectively.

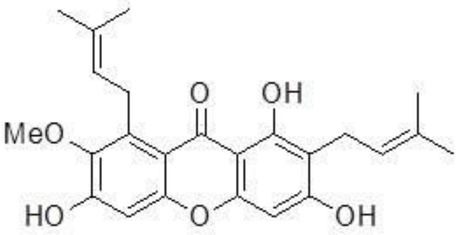

α–mangostin

α-mangostin (1 mg/ml-Retention time: 42.4 min)

Column: tsk-gel ODS-80Ts (TOSOH - 1 ml/min)
UV: 243 nm
Solvent: MeOH (A): water(B) - (A): 5%-5 min
(A): 5%-60%-10 min
(A): 60%-100%-35 min
(A): 100%-5%-1 min
(A): 5%-9 min

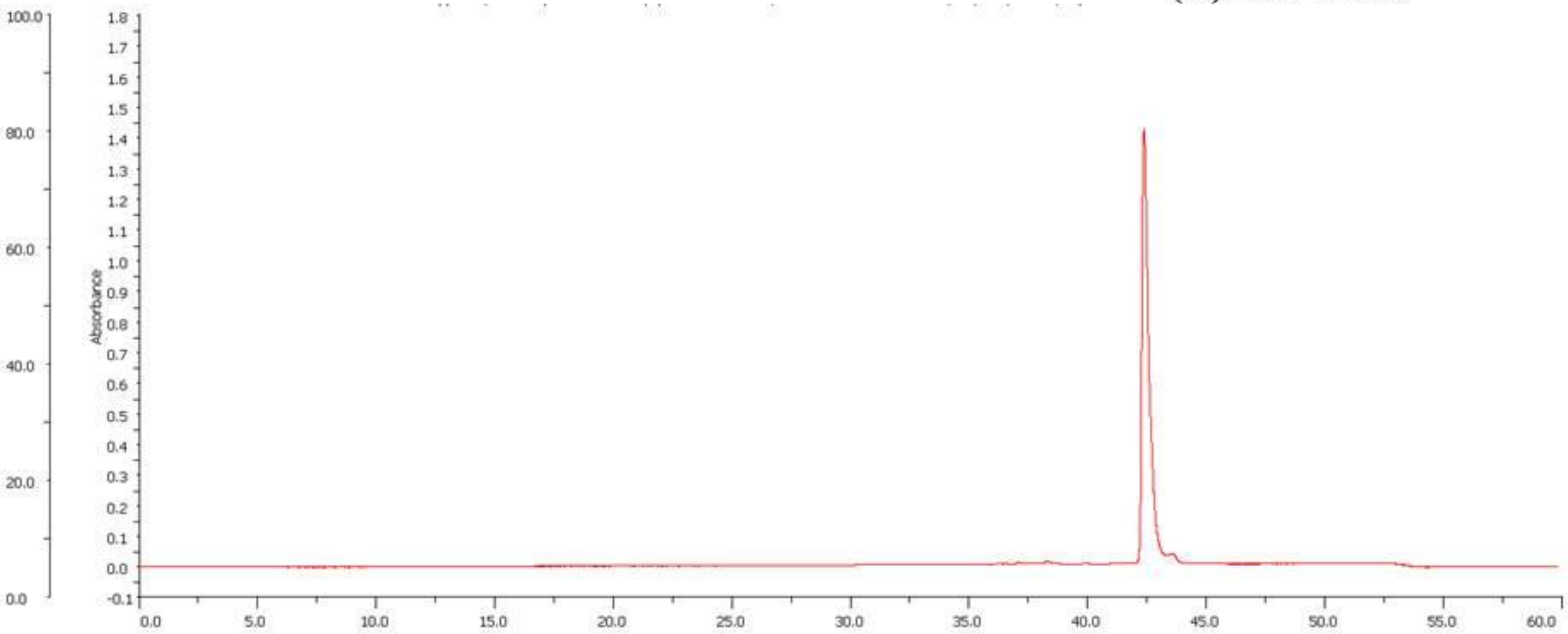

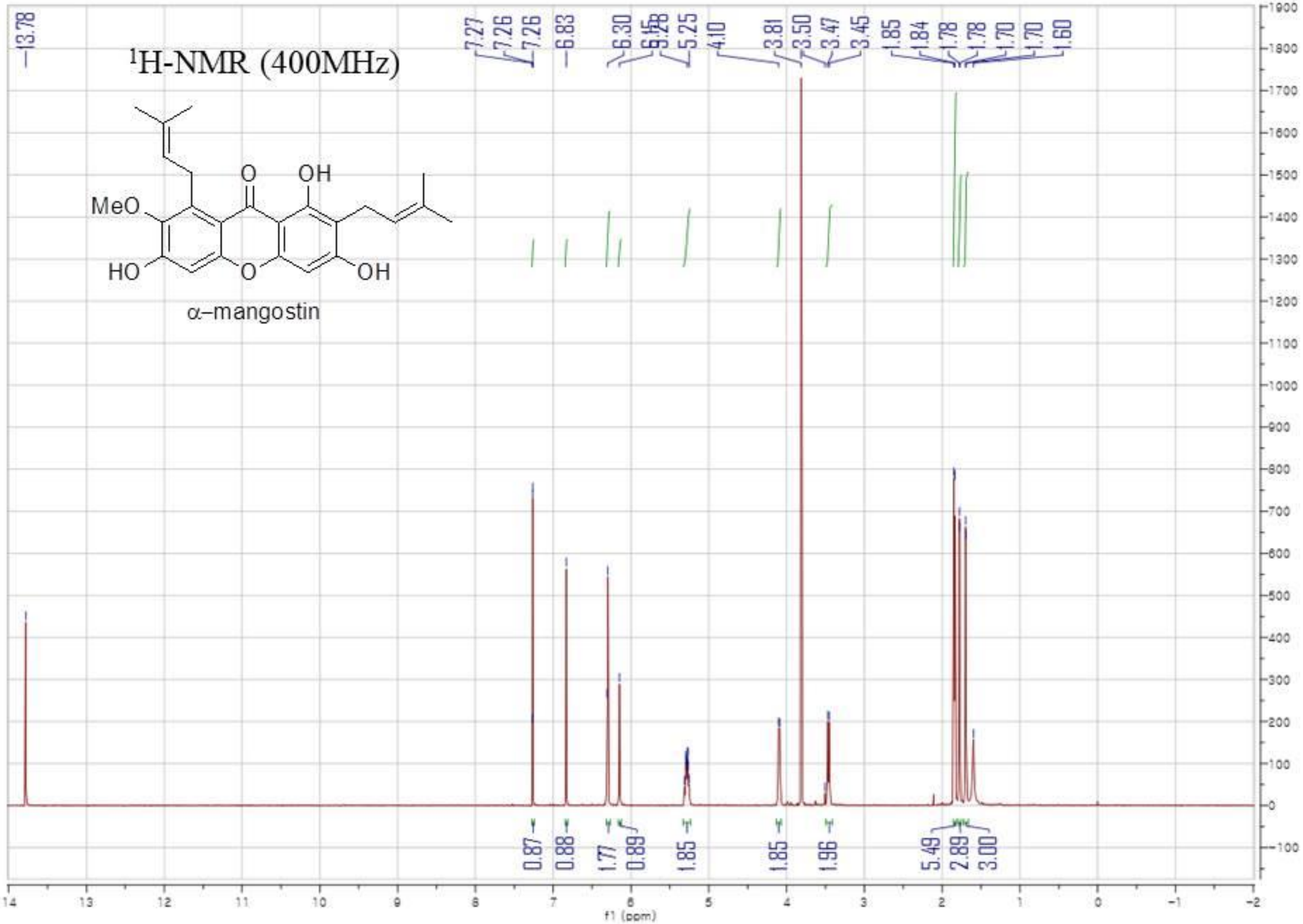

1H-NMR (400MHz)
MeO
O
OH
HO
O
OH
α–mangostin
13.78
7.27
7.26
7.26
6.83
6.30
5.25
4.10
3.81
3.50
3.47
3.45
1.85
1.84
1.78
1.78
1.70
1.70
1.60
0.87
0.88
1.77
0.89
1.85
1.85
1.96
5.49
2.89
3.00
f1 (ppm)

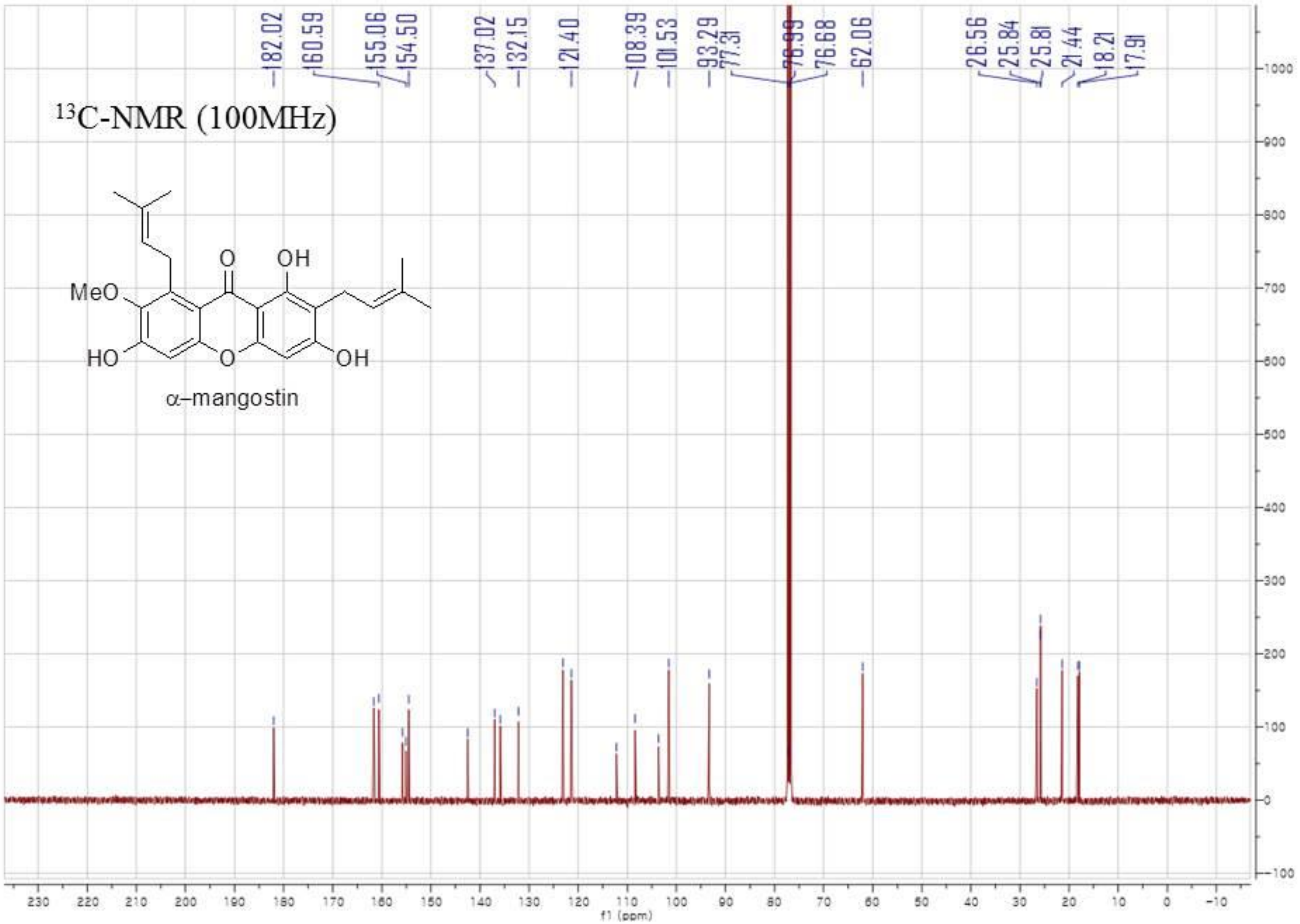

13C-NMR (100MHz)
182.02
160.59
155.06
154.50
137.02
132.15
121.40
108.39
101.53
93.29
77.31
76.99
76.68
62.06
26.56
25.84
25.81
21.44
18.21
17.91
MeO
O
OH
HO
OH
α–mangostin
f1 (ppm)

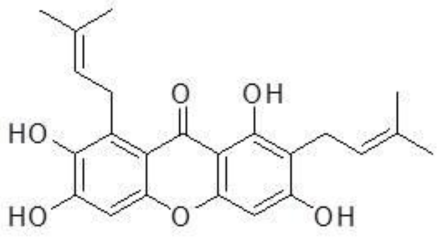

γ–mangostin

γ-mangostin (1 mg/ml-Retention time: 38.7 min)

Column: tsk-gel ODS-80Ts (TOSOH - 1 ml/min)
UV: 243 nm
Solvent: MeOH (A): water(B) – (A): 5%-5 min
(A): 5%-60%-10 min
(A): 60%-100%-35 min
(A): 100%-5%-1 min
(A): 5%-9 min

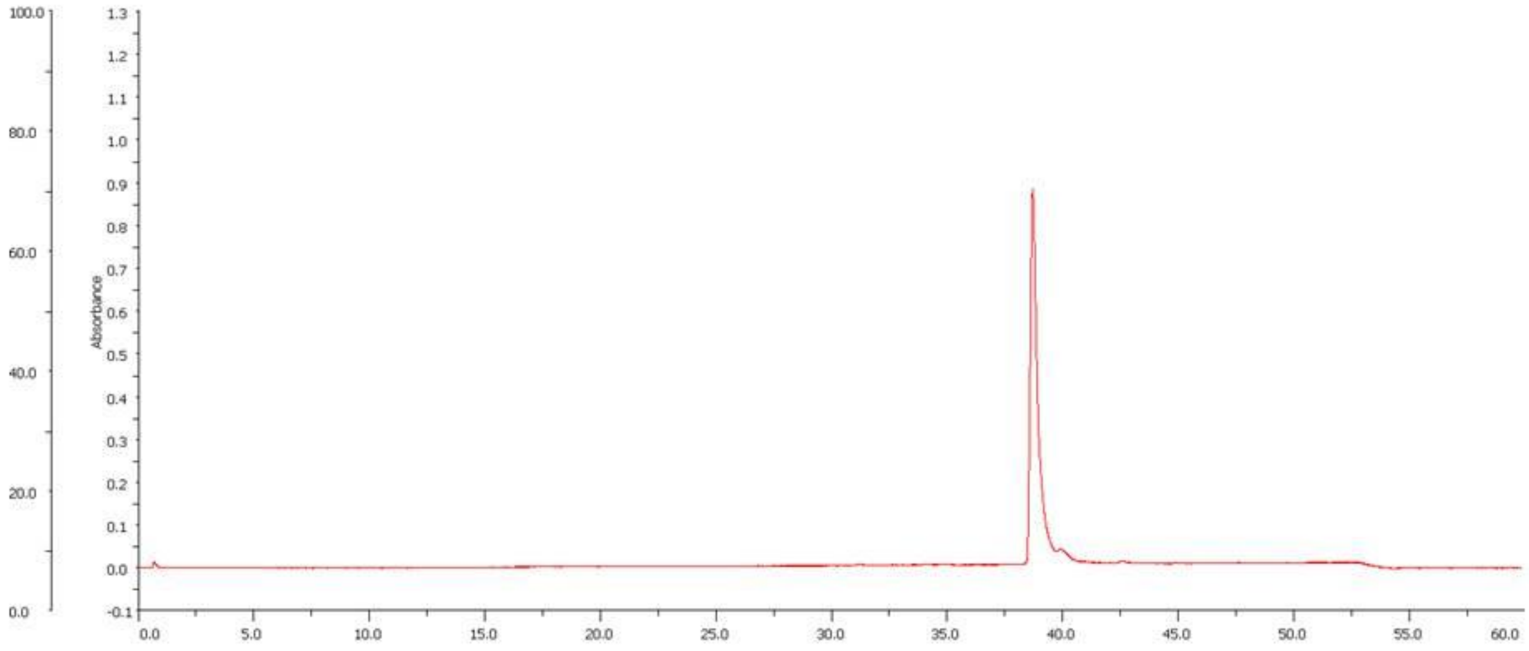

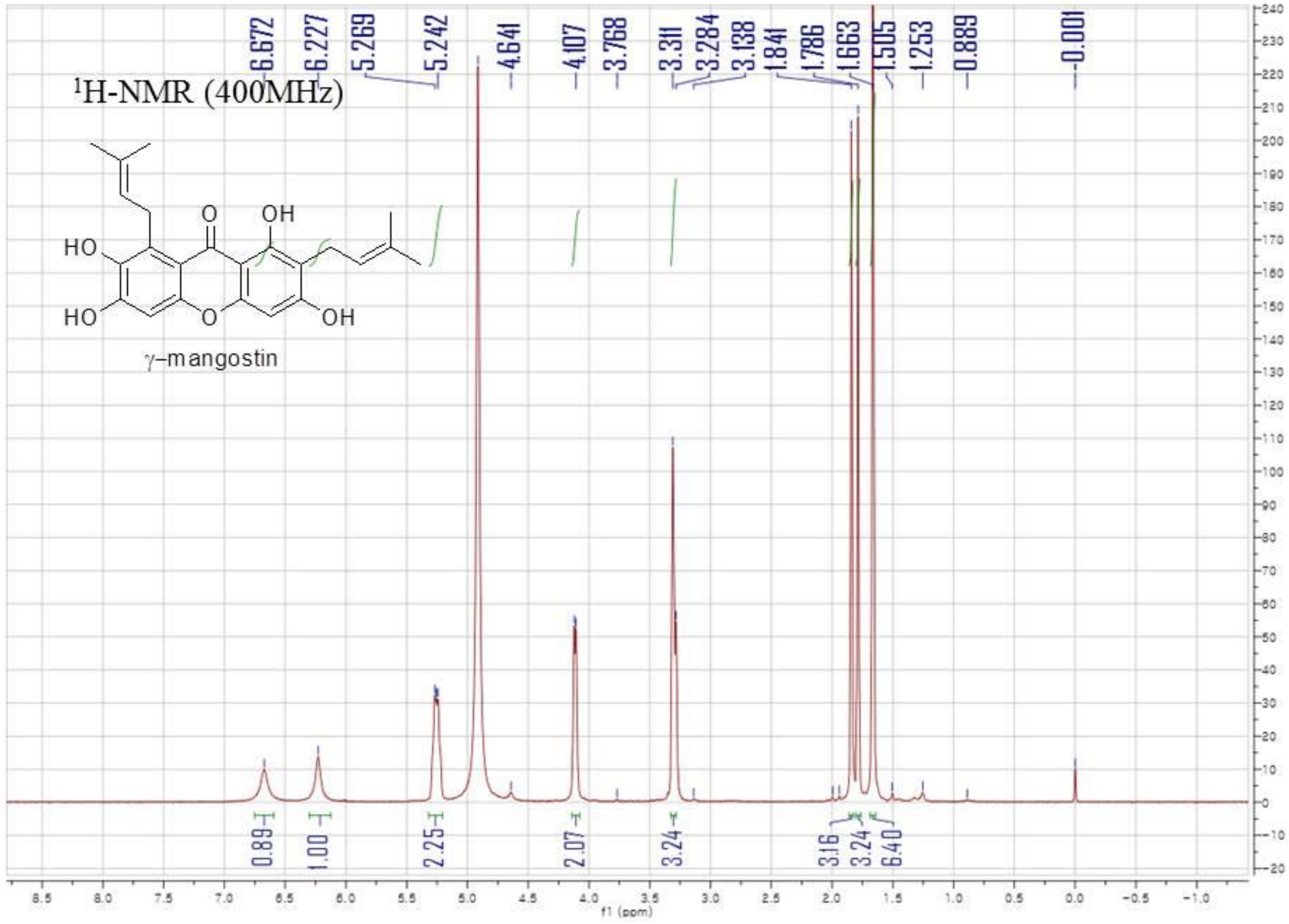

1H-NMR (400MHz)
γ–mangostin
OH
HO
HO
OH
O
O
6.672
6.227
5.269
5.242
4.641
4.107
3.768
3.311
3.284
3.138
1.841
1.786
1.663
1.505
1.253
0.889
-0.001
0.89
1.00
2.25
2.07
3.24
3.16
3.24
6.40
f1 (ppm)

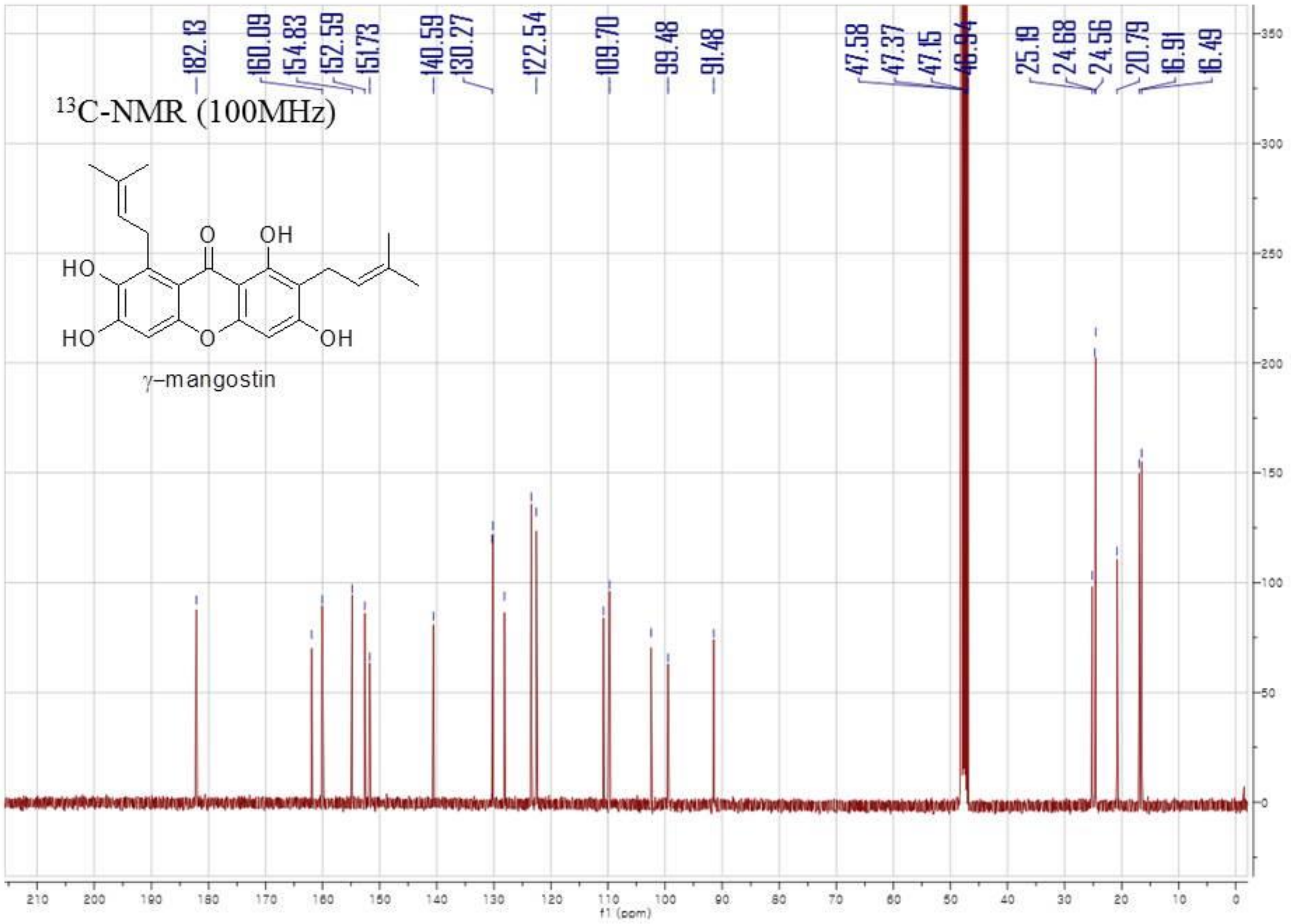

^{13}C-NMR (100MHz)
γ–mangostin
HO
HO
O
OH
OH
182.13
160.09
154.83
152.59
151.73
140.59
130.27
122.54
109.70
99.48
91.48
47.58
47.37
47.15
25.19
24.68
24.56
20.79
16.91
16.49
f1 (ppm)
